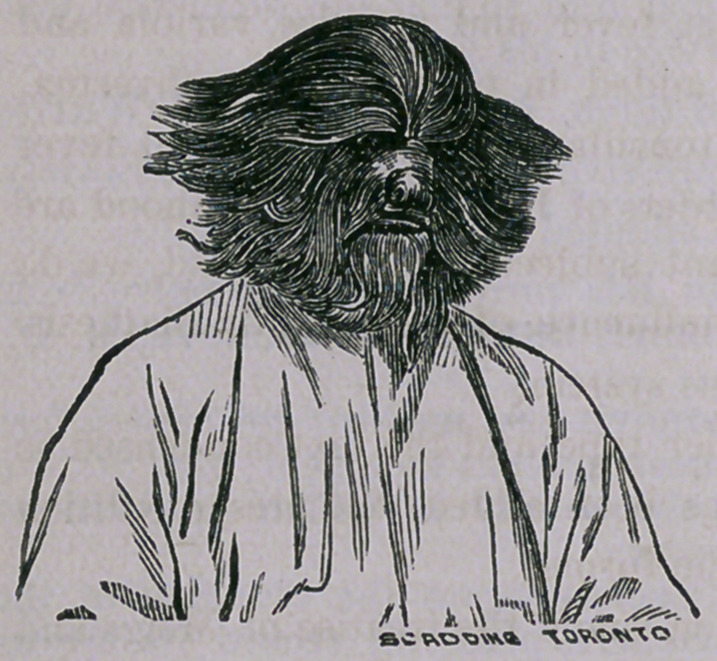# Hairy Man

**Published:** 1874-03

**Authors:** 


### Hairy Man.

The London *Lancet* of October, 25th gives the history and portrait of a Russian peasant and his son, Adrian and Fedor Jeftichjew, at that time on exhibition in Paris. We present below the picture of the father.

The entire face is covered with long brown hair, which extends down the back for some distance. An examination does not reveal any unhealthy condition of the skin, there being no nævoid or other discoloration. The son is only three years of age, and resembles his father in a marked degree, the hair, however, is lighter and thinner and the skin is therefore somewhat more perceptible. Both are nearly edentulous, the father having no teeth up to the age of seventeen, and then only four in the lower, and one in the upper jaw. The son has four incisors in the lower jaw. These are not the only known specimens of hairy men, other instances being mention as occurring among the Russians and Burmese. In these instances also an edentulous condition was onserved. The Hairy men of Japan are said not to resemble these, either in the absence of teeth or in the arrangement of hair.

These curious creatures excited considerable interest while in Berlin, and were there carefully examined by Prof. Virchow.

Whether they form a type of the connecting link between the present race of men and the previous lower order we cannot say, this and similar questions we leave for the consideration of Mr. Darwin. The picture reminds us forcibly of a Scotch Terrier, with a boy's coat on.

## Figures and Tables

**Figure f1:**